# Psychotherapy effectiveness for major depression: a randomized trial in a Finnish community

**DOI:** 10.1186/s12888-016-0838-1

**Published:** 2016-05-06

**Authors:** Hannu P. Saloheimo, John Markowitz, Tuija H. Saloheimo, Jarmo J. Laitinen, Jari Sundell, Matti O. Huttunen, Timo A. Aro, Tuitu N. Mikkonen, Heikki O. Katila

**Affiliations:** Hospital District of Helsinki and Uusimaa, Psychiatric Unit, Lohja subdistrict, Finland; New York State Psychiatric Institute, Columbia University, Markowitz, New York USA; Hospital District of Helsinki and Uusimaa, Helsinki University Hospital, Tuija Saloheimo, Finland; Numos OY, Espoo, Finland; Department of Psychiatry, Medical Faculty, Helsinki University, Huttunen, Finland; Department of Psychiatry, Ilmarinen Mutual Insurance Company, Helsinki University, Aro, Finland

**Keywords:** Depression, Randomized controlled trial, Effectiveness, Interpersonal Psychotherapy, Psychoeducative Group Therapy

## Abstract

**Background:**

The purpose of this study is to assess the relative effectiveness of Interpersonal Psychotherapy (IPT), Psychoeducative Group Therapy (PeGT), and treatment as usual (TAU) for patients with Major Depressive Disorder (MDD) in municipal psychiatric secondary care in one Finnish region.

**Methods:**

All adult patients (*N* = 1515) with MDD symptoms referred to secondary care in 2004-2006 were screened. Eligible, consenting patients were assigned randomly to 10-week IPT (*N* = 46), PeGT (*N* = 42), or TAU (*N* = 46) treatment arms. Antidepressant pharmacotherapy among study participants was evaluated. The Hamilton Depression Rating scale (HAM-D) was the primary outcome measure. Assessment occurred at 1, 5, 3, 6, and 12 months. Actual amount of therapists’ labor was also evaluated. All statistical analyses were performed with R software.

**Results:**

All three treatment cells showed marked improvement at 12-month follow-up. At 3 months, 42 % in IPT, 61 % in PeGT, and 42 % in TAU showed a mean ≥50 % in HAM-D improvement; after 12 months, these values were 61 %, 76 %, and 68 %.

Concomitant medication and limited sample size minimized between-treatment differences. Statistically significant differences emerged only between PeGT and TAU favoring PeGT. Secondary outcome measures (CGI-s and SOFAS) showed parallel results.

**Conclusion:**

All three treatments notably benefited highly comorbid MDD patients in a public sector secondary care unit.

**Trial registration:**

ClinicalTrials.gov NCT02314767 (09.12.2014).

## Background

Population surveys in Finland have found a 5 % prevalence of major depressive disorder (MDD), comprising more than 200,000 Finnish adults. An additional 15 % report milder depressive symptoms [1]. The prevalence of depressive disorders in Finland resembles that in other European countries and the United States [2, 3]. Depressive disorders are inversely associated with general well-being and health, cause serious psychosocial and work impairment, and produce a substantial societal burden [4]. Depression is the most costly psychiatric disorder in Europe, accounting for 33 % of the total cost of brain disorders. This corresponds to 1 % of the total European economy (GDP) [5]. Studies have found a temporal relationship between depression and functional disability [6].

Effective treatment can alleviate suffering and reverse the social and occupational decline of depressed patients. Several treatment modalities have shown efficacy in randomized clinical trials for MDD, including pharmacotherapy, interpersonal psychotherapy (IPT), cognitive-behavioral therapy, and behavioral activation therapy. Despite clinical practice guidelines issued to improve the quality of antidepressant care, studies have repeatedly shown that a high proportion of depressed Finnish patients receive suboptimal treatment. Only 10.6 % of depressed patients have ever received weekly psychotherapy during their treatment period [7, 8]. Treatments with established efficacy in highly controlled clinical trials need testing with randomized effectiveness trials in naturalistic settings to see how they work “in real life” [9].

Interpersonal psychotherapy (IPT) [10–12] and psychoeducational group therapy (PeGT) [13, 14] have demonstrated efficacy as monotherapies. In this study we aimed to test, in public mental health settings in the Lohja district of Finland, the effectiveness of these two structured, focused, time-limited antidepressant treatment modalities (IPT, PeGT) relative to treatment as usual (TAU). Finnish mental health services typically treat highly comorbid MDD patients. We hypothesized that IPT and PeGT would be more effective and would consume less time and fewer resources than would standard interventions. Given random allocation to conditions and consistent treatment protocols for all participants in terms of pharmacotherapy we assumed equal effectiveness of antidepressants in each treatment cell.

## Methods

Study recruitment lasted from February 2004 through March 2006.

Medline and PsychINFO surveys indicate that this is the largest randomized clinical trial of brief psychotherapies for patients with MDD and comorbid anxiety disorders in Scandinavia.

### Statistical analysis

Power analyses indicated group sample sizes of 32 were needed to achieve a 91 % power to detect a 4 point difference (assuming a pooled standard deviation of 4.0) in mean HAM-D scores from baseline to 3-month follow-up between groups. An alpha level of .01 for a two-tailed test was used in sample size calculations. To allow for attrition, however, the aim was to enroll a total of 140-160 patients (about 50 patients per group).

Baseline differences in demographic variables and the depression measurements across the three treatment groups were tested using chi-square tests, Kruskal-Wallis test and 1-way analysis of variance. To comply with the intention-to-treat principle in a setting with high dropout rates, likelihood-based inference using linear mixed models was employed to analyze treatment effects. The main focus in mixed models was on the treatment-time interaction (=the effectiveness of intervention). Separate analyses assessed IPT-TAU, PeGT-TAU, and IPT-PeGT comparisons. The modifying effect of baseline depression severity and use of medication at baseline on the effectiveness of intervention was tested using modifier-treatment-time interaction. Unstructured covariance structure was used in mixed models to account for the correlation between repeated measurements. Only patients with baseline measurement and at least one follow-up visit were included in longitudinal analysis. All statistical analyses were performed using R software [15].

### Patient selection

All adult Finnish-speaking patients, ages 18-64, whose referral documents to the Lohja and Vihti clinics noted depressive symptoms, were invited to participate in a diagnostic interview. The Lohja region, 60 km from Helsinki, encompasses two cities and four rural counties; its 79,000 inhabitants are two-thirds urban and one-third rural. Two clinics provide the area’s mental health outpatient services: the Lohja outpatient clinic and the Vihti outpatient clinic. The study teams worked equally in both clinics to ensure access for patients. All area referrals were funneled to the nearest clinic for assessment and treatment. Referrals came from local primary health care, occupational health care, and private general practitioners. One study psychiatrist (H.S.) conducted the screening assessments. Mental health outpatient clinic treatment is provided gratis for all patients in Finland.

### Diagnostic assessments

Patients had to fulfill ICD-10 diagnostic criteria [16] for unipolar depression (F32-F33) and meet suitability requirements for outpatient treatment. Diagnoses were verified by the Diagnostic and Statistical Manual of Mental Disorders 4th Edition (DSM-IV; [17]) utilizing the Finnish translation [18] of the Mini International Neuropsychiatric Interview (M.I.N.I.). Psychotic or severely suicidal patients needing hospitalization were excluded, as were patients with alcohol dependence or illicit substance abuse; however, patients were not excluded for anxiety disorders, alcohol harmful use or for stable, chronic somatic illness. Patients with serious acute somatic illness were referred elsewhere for appropriate treatment. Mental retardation was not an exclusion criterion, but inability to read and write was. Patients received a routine somatic check-up with basic laboratory tests including thyroid function.

Trained doctors (four psychiatrists and one resident) who were blinded to treatment assignment, treatment condition for the post-treatment and follow-up assessments served in the role of assessors and conducted the diagnostic interviews and administered 17-item Hamilton Rating Scale for Depression (HAM-D, [19]), the Social and Occupational Functioning Assessment Scale (Sofas, [20]), and the Clinical Global Impression of Severity and Change (CGI-s, CGI-c; [21]).

Depressive severity was assessed using the HAM-D [19], with an inclusion threshold of ≥14 points. This was the primary study outcome variable. The research protocol was approved by the Ethics Committee of Hospital District of Helsinki and Uusimaa, Finland, in compliance with the Declaration of Helsinki (approval number 20.1.2004/Dnro 517/E7/03).

After explaining the study protocol to patients, research staff asked them for their written informed consent. Patients also received written information about the study. Before randomization, those who had consented to participate were assessed for possible need for antidepressant medication or sick leave, according to guidelines from the Instructions Regarding Depression: Current Care Guideline, 2004 www.kaypahoito.fi [22]. Although IPT and PeGT have demonstrated antidepressant efficacy as monotherapies, the Current Care Guideline for Depression recommends antidepressant medication in moderate to severe ICD-10 depression. It recommends psychotherapeutic interventions for mild and moderate depression, but not as first-line treatment for severe depression. Therefore, because TAU includes antidepressant medication, clinicians recommended such medication to all study patients. Not all patients accepted this recommendation or the offer of sick leave. National Health Insurance Records were used to verify medication purchases.

Screening and assessment personnel did not treat study patients. The human resources expended for each treatment modality was assessed by number of visits in each group and hours of actual therapy.

### Treatment trial

Patients entering the study were randomized to three treatment arms: 1) IPT, 2) PeGT, or 3) TAU. IPT and PeGT were alternatives to TAU. All patients were recommended antidepressant pharmacologic treatment. A computer program randomly allocated 134 patients equally across the treatment groups. Codes were kept in separate, opaque envelopes, opened post-consent by the research assistant, who then assigned patients to the proper treatment protocol. The IPT treatment team comprised two psychiatric nurses and one social worker; the PeGT team had one psychologist and three psychiatric nurses. TAU was conducted by other outpatient clinic staff members of comparable experience. Assessors were blinded to treatment modality. Patients were instructed not to describe their treatment to the raters.

### Treatments

1. IPT, a time-limited, manualized [23, 24] psychotherapy, focuses on current interpersonal problems. IPT reduces depressive symptoms and improves interpersonal functioning. It uses a medical model of depressive illness, relates symptoms to a focal interpersonal problem area (grief, role dispute, role transition, or interpersonal deficits), helps patients understand their affects as useful interpersonal environmental signals, and mobilizes social supports.
2. PeGT is a psychoeducational self-treatment of depression in group format. Therapists act as coaches for groups of 4-6 patients. Lewinsohn and colleagues created this treatment model and published the “Control Your Depression” protocol in 1986 [13]. Koffert and Kuusi translated this manual including a workbook into Finnish in 2002 [25].
3. TAU in Finnish public mental health clinics typically provides low-frequency individual supportive psychotherapy of variable duration conducted by a psychiatric nurse, and antidepressant medication from a psychiatrist who sees the patient in brief medication visits every 4-6 weeks. TAU personnel were advised to implement treatment as they usually would. The doctors who recommended sick leave and possible medication during the treatment phase were blinded to treatment modality and did not participate in treating the patients they rated.

The IPT and PeGT therapies comprised 10 therapeutic sessions. IPT usually comprises 12-16 weekly sessions but has been delivered in doses as low as 8 sessions [26, 27]. The 10-session approach was deemed suitable based on estimation that TAU provided a mean of 10 therapeutic sessions per patient, including pharmacotherapy sessions. The 10-session acute therapy was planned to occur over 10-12 weeks in IPT and PeGT, with more variable time and sessions in TAU. This acute treatment phase had a 12-month follow-up.

### Training

Three psychiatrists (two with over 15 years and one with over 7 years of experience) and one psychiatric resident (with over 5 years of experience) served as assessors. Training in standardized use of the M.I.N.I., HAM-D-17, CGI-c, and SOFAS included a lecture by Finnish experts and practical training including videotaped patient cases. Regular rater meetings maintained diagnostic and rating skills.

#### Therapists

The four IPT therapists were trained with a set of theoretical lectures and then completed three supervised pilot training cases. Supervision was executed by two psychiatrists both with over 5 years of experience in IPT treatment and supervision. Three psychiatric nurses and one clinical psychologist received training for PeGT by reading the protocol and taking the “Control Your Depression” course. They were led by psychiatrist who has over 5 years’ experience with this treatment and co-translated the book into Finnish [25]. Study personnel were recruited from the permanent clinic staff who had expressed interest in the study and had at least 5 years of clinical experience. They were blinded to treatment modality before recruitment and were assigned randomly to one of the therapy arms and then trained in IPT or PeGT. This procedure was intended to maximize acquisition of psychotherapists in this region. TAU therapists were advised to implement “routine” TAU. The mean age of IPT therapists was 45 years, and PeGT therapists, 44 years. All had more than 5 years’ experience in treating depressed patients. TAU therapists had similar experience.

Both treatment modalities continued group supervision monthly throughout the trial. For PeGT, patient completion of the workbook was considered adherence to the therapy model. For IPT, 40 % of therapy sessions were taped, and 20 % of the taped sessions were reviewed in therapist groups with subsequent commentary by the supervisor to confirm fidelity to the treatment. Therapist adherence was not formally measured.

### Assessment and follow-up

Assessments were conducted at baseline, 1.5 months, 3 months, 6 months and 1 year. The primary outcome point for effectiveness was immediately post-treatment (3 months).

A key objective was to assess effectiveness of the different treatments. Response was defined *a priori* as ≥50 % decrease in baseline HAM-D-17 score, and remission as HAM-D-17 score ≤7. Other comparisons included the Clinical Global Impression Severity and Improvement (CGI-s, CGI-I) and the Social and Occupational Functioning Assessment Scale (SOFAS).

IPT and PeGT patients attending at least 7 therapy sessions were considered completers. No such criterion was applied to the TAU group because of natural variability in TAU attendance. We counted the number of visits and actual personnel time contributed to therapy and consultations in each cell.

## Results

**Fig. 1:**
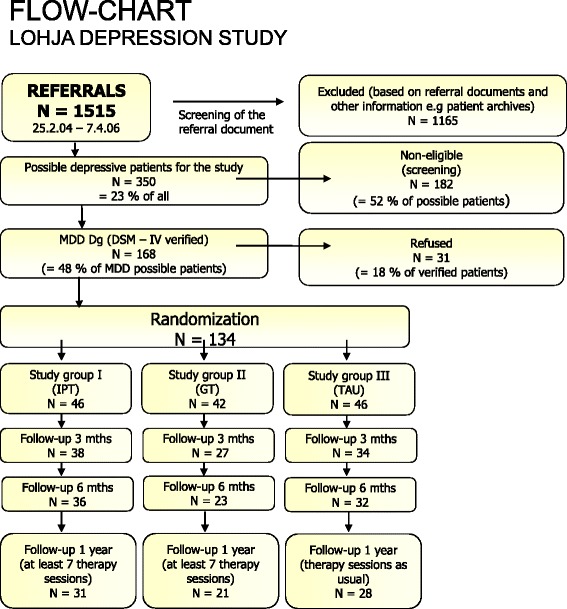
Referrals, pre-screening, screening, randomization, and attrition in the trial

Figure 1 shows the patients selection, randomization and attrition of the 134 patients in this study. Of 165 patients with MDD diagnoses subsequently verified by M.I.N.I., 31 (19 %) refused study participation (Table 2). The 134 remaining patients were randomized to the three study groups IPT group consisting 46, GT group 42 and TAU group 46 patients at baseline (Fig. 1). In IPT, 31 (67 %) patients completed the 12-month follow-up, compared with 21 (50 %) in PeGT and 28 (61 %) in TAU (χ^2^(2) = 2.80, *p* = .25).

The mean number of total visits for psychiatric treatment was 12.5 (SD 3.4) in IPT, 11.1 (SD 2.5) in PeGT, and 14.6 (SD 9.3) in TAU. No statistically significant differences appeared across the study groups (F(2,77) = 2.01, *p* = .14). We lacked access to data from services outside the public sector (e.g., health centers, private physicians).

Median personnel time spent on psychotherapy or TAU counseling among study completers did not differ significantly by group: 10.0 (IQR 1.0) hours for IPT, 12.1 (IQR 4.9) hours for PeGT, and 12.0 (IQR 11.5) hours for TAU (χ^2^(2) = 0.78, *p* = .68, Kruskal-Wallis test). PeGT was implemented in nine groups (mean 3.47 patients per group) to minimize delay, with two therapists conducting the 2-hour sessions.

### Sample demographics

Baseline analyses revealed no significant group differences in age, sex or marital status (Table 1). PeGT patients had significantly higher employment level than TAU patients (χ^2^(1) = 7.98, *p* = .005).

**Table 1:** Baseline clinical and demographic characteristics of the experimental groups

Characteristics	IPT (*N* = 46)	PeGT (*N* = 42)	TAU (*N* = 46)	χ^2^ or F	DF	P-value*
Sex				2.02	2	.36
Male	16 (35 %)	11 (26 %)	10 (22 %)			
Female	30 (65 %)	31 (74 %)	36 (78 %)			
Job situation				8.08	2	.018
Employed	36 (78 %)	38 (90 %)	30 (65 %)			
Not employed^1^	10 (22 %)	4 (10 %)	16 (35 %)			
Marital status				1.63	2	.44
Spouse	30 (65 %)	25 (60 %)	24 (52 %)			
No spouse	16 (35 %)	17 (40 %)	22 (48 %)			
Mean Age	43 (12.3)	42 (10.1)	40 (125)	0.93	2/131	.40
Mean HAM-D (SD)	18.7 (4.0)	19.3 (3.8)	18.9 (3.7)	0.31	2/131	.74
Mean CGI (SD)	4.5 (1.1)	4.5 (1.2)	4.6 (0.8)	0.08	2/131	.92
Mean JES^2^ (SD)	30.4 (6.1)	31.3 (6.7)	30.6 (6.7)	0.13	2/104	.88
Mean SOFAS (SD)	53.7 (9.5)	54.9 (10.3)	53.2 (8.8)	0.38	2/131	.68
Mean 15-D^3^ (SD)	0.73 (0.09)	0.76(0.07)	0.74 (0.09)	1.04	2/101	.36

* = p-value for the overall difference among treatment groups

DF = Degrees of freedom for χ^2^-test or 1-way ANOVA (DF_num_/DF_den_)

^1^ = includes housewives, students, unemployed, non-military servants, and laid-off individuals

^2^ = 27 missing values

^3^ = 30 missing values

### Refuser demographics

Comparisons of demographic characteristics of study refusers with study enrollees revealed statistically significant differences on HAM-D scores and age: study refusers were younger and slightly less depressed (mean difference 1.6 HAM-D points) (Table 2).

**Table 2:** Refuser characteristics

Characteristics	Total	Refusers	χ^2^ or t	DF	P-value
Sex			0.33	1	.57
Male	37 (28 %)	7 (23 %)			
Female	97 (72 %)	24 (77 %)			
Job situation			0.62	1	.43
Job	104 (78 %)	22 (71 %)			
No job	30 (22 %)	9 (29 %)			
Marital status			1.53	1	.22
Spouse	79 (59 %)	22 (71 %)			
No spouse	55 (41 %)	9 (29 %)			
Mean HAM-D (SD)	18.9 (3.8)	17.3 (2.5)	3.70	34	.0008
Mean age (SD)	41.9 (11.7)	36.3 (11.9)	2.37	45	.022

### Comorbidity

Summing comorbid diagnoses for any anxiety disorder and substance-related disorders revealed no statistically significant differences between study patients and refusers. Comorbidity rates for any anxiety disorder were 57 % among enrollees vs. 58 % among study refusers (χ^2^(1) = 0.00, *p* = .95); and 59 % in IPT, 55 % in PeGT, and 56 % in TAU (χ^2^(2) = 0.14, *p* = .93). Median AUDIT scores measuring alcohol and substance abuse were 3.0 (IQR 7.0) for IPT, 3.0 (IQR 6.0) for PeGT, and 4.0 (IQR 5.5) for TAU (χ^2^(2) = 0.44, *p* = .80, Kruskal-Wallis test). In AUDIT scoring, 4 of 40 points indicates elevated risk for harmful use of alcohol in men; for women, the cutoff is 3 of 40.

### Pharmacotherapy for depression and patient treatment attendance

We documented antidepressants and adjunct pharmacologic therapy in all study groups. This was performed by using doctors’prescriptions and National Health Insurance statistics to clarify compliance. In all three study groups there were patients who refused pharmacotherapy.(We did not attempt blood-level testing.) Adequate dosage was defined by Käypä Hoito (Finnish Guidelines for the treatment of Patients with Depression). The Guidelines recommend antidepressant pharmacotherapy to patients who are suffering moderate to severe depression according to ICD-10 criteria. All patients in this study were recommended to receive antidepressant treatment. Doctors’ prescriptions also followed the Guidelines. Two patients in each cell used quetiapine and risperidone as adjuvant pharmacotherapy. We found no statistically significant differences concerning types or combinations of antidepressants.

We divided the groups according to antidepressant medication use during the treatment and 12-month follow-up phases: 1) patients not using antidepressants; 2) patients who filled one to three monthly prescriptions, considered a proxy for suboptimal pharmacological adherence; and 3) patients who filled four or more prescriptions, considered a proxy for substantial pharmacologic intervention. We assumed that monthly prescriptions contained adequate antidepressant medication for the period.

We measured antidepressant use by study patients at 3 months and 1 year, and by study completers over 1 year.

We found no statistically significant differences between study groups at 3 months (χ^2^ (4) = 2.61, *p* = .63) or at 1 year (χ^2^(4) = 5.96, *p* = .20); however, there were statistically significant differences among study completers (Table 3): Four or more purchased packages which was considered as substantial pharmacological adherence were seen by 21 (91 %) patients in PeGT, 24 (67 %) in IPT and 16 (50 %) in TAU groups (χ^2^(4) = 13.92, *p* = .007 for the overall differences among treatment groups).

**Table 3:** Antidepressant use of study completers, by group

Number of prescriptions filled	IPT	PeGT	TAU
	N (%)	N (%)	N (%)
0	7 (19 %)	2 (9 %)	5 (16 %)
1-3	5 (14 %)	0 (0 %)	11 (34 %)
4 or more	24 (67 %)	21 (91 %)	16 (50 %)

χ^2^ = 13.92, df = 4, *p* = .007 for the overall differences by treatment group

### Dropout

We studied retention rate and reasons for attrition. During the 12-month follow-up, dropout rates were 33 % in IPT, 50 % in PeGT and 39 % in TAU groups (χ^2^(2) = 2.80, *p* = .25). Five patients in IPT, eight in PeGT and seven in TAU dropped out after baseline measurement without any follow-up visits (χ^2^(2) = 1.16, *p* = .56). One patient in IPT, six in PeGT and none in TAU dropped out after randomization but before the first treatment session. Reanalysis of the data with an intent-to-treat principle including these patients in longitudinal analysis did not change the findings. Reason unknown (unable to contact patient) was the most common attrition occurrence, applying to eight IPT, six PeGT, and five TAU patients. Psychiatric hospitalization or clinical deterioration explained three IPT, two PeGT, and five TAU cases who were classified as non-responders. Five patients in PeGT, one in IPT, and two in TAU dropped out because of subjective dissatisfaction, as reported in freely written comments. Three IPT patients and three TAU patients had incomplete follow-up because they relocated outside the region. Two TAU patients reported lack of motivation for treatment. Five PeGT patients sought individual therapies after study intervention; these therapies consisted of family counseling or private, individual psychodynamic-oriented psychotherapy.

## Outcome

**Fig. 2:**
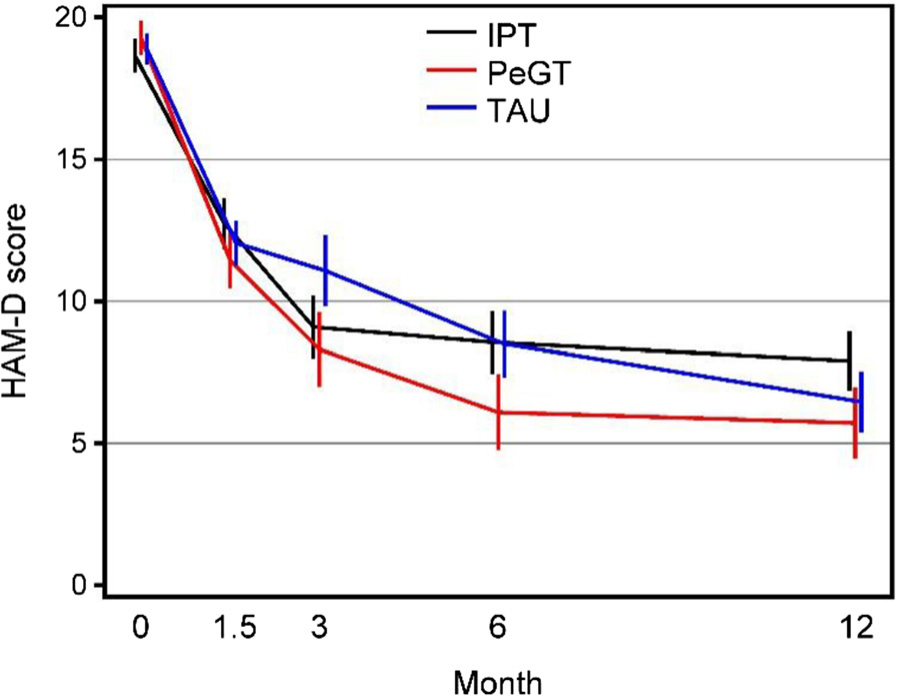
Mean (standard error of the mean) 17-Item Hamilton Depression Rating scale scores by treatment group at the baseline, and 1.5-, 3-, 6-, and 12-month follow-up

Patients were categorized according to their HAM-D scores at baseline as having moderate (≤20) or severe (≥21) depression. The modifying effect of baseline depression severity (≤20 or 21≥) on the effectiveness of intervention was tested using a linear mixed model. No significant interaction effects of depression severity, treatment, and time on HAM-D (F(8,108) = 1.11, *p* = .36), SOFAS (F(8,108) = 0.57, *p* = .80) or CGI (F(8,108) = 0.68, *p* = .71) were detected. Similarly, no significant interactions of time, treatment, or use of medication at baseline on HAM-D (F(8,108) = 0.87, *p* = .54), SOFAS (F(8,108) = 0.25, *p* = .98), or CGI (F(8,108) = 0.51, *p* = .85) were detected. All treatment groups improved rapidly. Immediately post-treatment, mean HAM-D score reductions were 9.1 (SD 6.4) for IPT, 8.3 (SD 6.3) for PeGT, and 11.1 (SD 6.4) for TAU. The time-treatment interaction in HAM-D score changes approached statistical significance at 3, 6, and 12 months (F(4,184) = 2.23, *p* = .067; F(6,263) = 1.74, *p* = .11, and F(8,340) = 1.68, *p* = .10, respectively) (Fig. 2). PeGT was significantly more effective than TAU at 3, 6, and 12 months (time-treatment interaction F(2,116) = 3.52, *p* = .033, F(3,162) = 2.91, *p* = .036, and F(4,209) = 2.76, *p* = .029, respectively). No significant difference in effectiveness was detected between IPT and TAU, nor between IPT and PeGT. When linear mixed models controlled for cumulative number of filled prescriptions, the time-treatment interaction was significant at 12 months (F(8,338) = 1.99, overall *p* = .046; PeGT vs TAU F(4,207) = 3.84, *p* = .0049; IPT vs TAU F(4,244) = 1.84, *p* = .12). Because the number of filled prescriptions was not a significant factor for HAM-D scores in mixed models, it was removed from the final models.

All three groups showed over a 50 % mean decrease in HAM-D scores from baseline to 6 months. At 3 months, IPT and TAU showed a slightly (nonsignificantly) greater decrease. Remission rates did not differ for any follow-up interval: 42.4 % for IPT, 60.9 % for PeGT, and 42.3 % for TAU s (χ^2^(2) = 2.27, *p* = .32 for overall difference) at 3 months; 58.8, 65.0, and 50.0 % (χ^2^(2) = 1.13, *p* = .57 for overall difference) at 6 months; 51.6, 71.4, and 64.3 % (χ^2^(2) = 2.24, *p* = .33 for overall difference) at 12 months. The high dropout rate, especially in the PeGT (50 %) and TAU (39 %) groups, lowered statistical power.

No differences were found in effectiveness of treatment groups on SOFAS (time-treatment interaction, F(8,338) = 0.56, *p* = .81) or on CGI (time-treatment interaction, F(8,336) = 0.44, *p* = .90) during the 12-month follow-up. No subject died or attempted suicide during follow-up.

## Discussion

The study describes outcomes for three treatment modalities for depressed (MDD) patients in specialized care in a naturalistic, public sector setting. To our knowledge, this is the largest psychotherapy effectiveness RCT for major depressive disorder in the Nordic countries. We hypothesized that well-organized, structured, and focused time-limited psychotherapies would be more effective and consume less resources than does the standard intervention.

Results indicate that patients improved markedly and quickly in all three treatment cells and maintained gains at 12-month follow-up. Reassuringly, patients in the Finnish public mental health system responded to standard antidepressant treatment; in fact, these patients fared so well that addition of IPT and PeGT to the standard pharmacotherapy options produced only a small further advantage. This suggests a ceiling effect: there was little further room to show improvement (Fig. 2). Some of that additional advantage may reflect the fact that the psychotherapy interventions seemed to improve medication adherence (Table 3).

The study maximized internal validity through diagnostic tools and randomization. It included high anxiety disorder comorbidity in an externally valid, “real life” setting, a municipal mental health secondary level unit. Referrals were screened by psychiatrists, and assessors were trained thoroughly to use the diagnostic instruments. All treatments showed a 50 % or greater decrease from baseline to 6 months, an outcome well within the range of response in efficacy trials [28]. A statistically significant difference emerged only between PeGT and TAU groups and favored PeGT at follow-up time 3-months to 12-months. This result partly supports the primary hypothesis of effectiveness. The results encouraged a closer examination of the study population and study personnel, the training procedure, quality control of the treatment implementation, and other factors that could influence the outcome. First: standard therapies seem highly effective in the Finnish public mental health system, particularly in light of the fact that most patients suffering from MDD do not receive weekly psychotherapy. Far from an inert control, TAU was an active, potent comparator. In fact, there is growing criticism over the use of TAU as a control comparator in clinical trials [29, 30]. Burns [29] states that using TAU obscures findings; furthermore, he asserts that the problem begins with considering TAU as a control rather than an equally or even more resource-demanding treatment. TAU in this study seems not to have been a minimal intervention. Treatment “contamination” may have occurred; e.g., aspects of IPT and PeGT may have spilled over inadvertently on public health ambulatory units where some personnel used specific treatments while others employed treatment as usual for depressed patients.

Second: Preference differences may have played a role. PeGT had the greatest attrition rate. Some patients, having been randomized to PeGT, did not want to participate in a group treatment, and others left after one or two sessions, feeling that group treatment did not suit them. Other study subjects may have found group treatment more attractive and motivating. This secondary post-randomization self-selection might have influenced the results in favor of PeGT. Conversely, PeGT patients who dropped out may have been less likely to improve than those who remained, so the former patients’ discontinuation could have caused inflation of results for this group. Unfortunately, we did not assess patient preferences for treatment.

Third: Variable use of antidepressant medication may also have complicated the outcome results. Therapists working in psychiatric ambulatory secondary care tend to have quite positive attitudes towards pharmacologic treatment. In PeGT groups this attitude may have encouraged patients to accept and maintain antidepressant treatment. PeGT study completers were more likely to use antidepressant pharmacotherapy in adequate duration and doses than were the other study modalities. The group format seemed to yield better treatment compliance with both psychotherapy and pharmacotherapy, for patients who did not drop out.

Given the widespread use of antidepressant medication in the treatment sample, this study appears underpowered to find differences among the psychotherapies used.

Additionally, variable adherence by therapists may have influenced the results. Monthly supervision of structured therapeutic interventions is likely too infrequent for accurate monitoring of adherence, this also may have influenced the results. Therapists were randomly assigned to structured treatments: all personnel in ambulatory units were interviewed and taught therapeutic tools, but therapists could not choose the therapy they were asked to implement. This may have influenced therapeutic allegiance. However, when interviewed afterwards, all therapists reported satisfaction with their treatment and said they still used the approach in their everyday work.

Treatment attendance evaluation showed that TAU group patients used non-significantly more services during follow-up than did the structured treatment group patients, consistent with one of our primary hypotheses.

One strength of time-limited therapies is that they encourage patients to regain remission. This study design feature may have positively influenced the frequency and coherent planning of TAU and thus minimized the differences between study groups while being of benefit to all study participants.

The study has several limitations. The high attrition rate very likely influenced the results. We did not evaluate patient treatment preferences at baseline, even though preference often has been found to moderate treatment results [31]. We did not evaluate therapist preference but randomly assigned therapists to therapy models. This study is underpowered to find differences among psychotherapies because of the concomitant use of antidepressant medication. Furthermore, interrater reliability was not assessed formally.

## Conclusions

In this research we studied MDD patients with high comorbidities in three treatment modalities. According to the primary outcome measure all patients benefited notably. Secondary outcome measures showed parallel results. Improvement maintained over the 12-month follow-up. To avoid statistical limitations future studies should be performed with larger samples and take into account both patients’ and therapists’ treatment preferences. Additionally, costs of antidepressant medication, as well as secondary costs of side effects of biological and psychotherapeutic interventions and of sick leave and diminished social and functioning capacity should be measured.

## Ethical approval and consent to participate

The Ethics Committee of Hospital District of Helsinki and Uusimaa approved the study and participants gave written-informed consent before participation.

## Consent for publication

Not applicable.

## Availability of data and materials

The data and materials used in this paper are available by contact with corresponding author.

## Abbreviations

CGI-s and CGI-c: Clinical Global Impression of Severity and Change
HAM-D: Hamilton Depression Rating Scale
IPT: Interpersonal Psychotherapy
M.I.N.I.: Mini International Neuropsychiatric Interview
MDD: Major Depressive Disorder
PeGT: Psychoeducative Group Therapy
SOFAS: Social and Occupational Functioning Assessment Scale
TAU: Treatment as usual
